# Unpacking public resistance to health Chatbots: a parallel mediation analysis

**DOI:** 10.3389/fpsyg.2024.1276968

**Published:** 2024-04-10

**Authors:** Xiqian Zou, Yuxiang Na, Kaisheng Lai, Guan Liu

**Affiliations:** ^1^School of Journalism and Communication, Tsinghua University, Beijing, China; ^2^School of Journalism and Communication, Jinan University, Guangzhou, Guangdong, China; ^3^Center for Computational Communication Studies, Jinan University, Guangzhou, Guangdong, China

**Keywords:** health chatbots, resistance behavioral tendency, innovation resistance theory, prototype willingness model, parallel mediation analysis, partial least squares structural equation modeling

## Abstract

**Introduction:**

Despite the numerous potential benefits of health chatbots for personal health management, a substantial proportion of people oppose the use of such software applications. Building on the innovation resistance theory (IRT) and the prototype willingness model (PWM), this study investigated the functional barriers, psychological barriers, and negative prototype perception antecedents of individuals’ resistance to health chatbots, as well as the rational and irrational psychological mechanisms underlying their linkages.

**Methods:**

Data from 398 participants were used to construct a partial least squares structural equation model (PLS-SEM).

**Results:**

Resistance intention mediated the relationship between functional barriers, psychological barriers, and resistance behavioral tendency, respectively. Furthermore, The relationship between negative prototype perceptions and resistance behavioral tendency was mediated by resistance intention and resistance willingness. Moreover, negative prototype perceptions were a more effective predictor of resistance behavioral tendency through resistance willingness than functional and psychological barriers.

**Discussion:**

By investigating the role of irrational factors in health chatbot resistance, this study expands the scope of the IRT to explain the psychological mechanisms underlying individuals’ resistance to health chatbots. Interventions to address people’s resistance to health chatbots are discussed.

## 1 Introduction

Health chatbots are revolutionizing personal healthcare practices (Pereira and Díaz, 2019). Currently, health chatbots are utilized for personal health monitoring and disease consultation, diagnosis, and treatment (Tudor Car et al., 2020; Aggarwal et al., 2023). For example, a virtual nurse named “Molly,” developed by researchers at the Maastricht University Medical Center+ (MUMC+), offers healthcare guidance to patients with heart disease (Zorgenablers, 2019), and chatbots such as “Youper” have been designed to track users’ mood and provide them emotional management advice (Mehta et al., 2021). Further, “Tess” is a mental health chatbot that provides personalized medical suggestions to patients with mental disorders (Gionet, 2018), similar to a therapist. Remarkably, a personal health assistant aimed at preventative healthcare, “Your.MD,” has thus far been used to provide diagnostic services and solutions to nearly 26 million users worldwide (Billing, 2020). According to BIS Research, the global market for healthcare chatbots is expected to reach $498.1 million by 2029 (Pennic, 2019).

Medical artificial intelligence (AI) services, including health chatbots, are expected to be crucial for promoting the quality of healthcare, addressing the inequitable distribution of healthcare resources, reducing healthcare costs, and increasing the level and efficiency of diagnosis (Guo and Li, 2018; Lake et al., 2019; Schwalbe and Wahl, 2020). However, more participants preferred consulting with doctors rather than health chatbots for medical inquiries (Branley-Bell et al., 2023), even if they operate with the same level of expertise as human doctors (Yokoi et al., 2021); a significant number of users drop out during consultations with health chatbots (Fan et al., 2021), with nearly 40% of the people unwilling to even interact with them (PWC, 2017). Notably, many specialists are worried about the inherent limitations relating to potential discriminatory bias, explainability, and safety hazards of medical AI (Amann et al., 2020). For instance, one survey found that over 80% of professional physicians believe that health chatbots are unable to comprehend human emotions and represent the danger of misleading treatment by providing patients with inaccurate diagnostic recommendations (Palanica et al., 2019). Further, people perceive health chatbots as inauthentic (Ly et al., 2017), inaccurate (Fan et al., 2021), and possibly highly uncertain and unsafe (Nadarzynski et al., 2023), leading to their discontinuation or hesitation in circumstances where medical assistance is required. Although overcoming public resistance to AI healthcare technologies is critical for promoting its societal acceptance in the medical field in the future (Gaczek et al., 2023), few studies have investigated how resistance behavior toward AI healthcare technologies (e.g., health chatbots) is formed. Therefore, the first research question of this study was to explore which factors influence people to resist health chatbots.

Resistance is a natural behavioral response to innovative technology, as its adoption may change existing habits and disrupt routines (Ram and Sheth, 1989). The delayed transmission of innovation in the early stages of its growth is primarily attributed to people’s resistance behavior (Bao, 2009). Previous research has primarily focused on the impact of rational calculations underlying individuals’ technology adoption/resistance intentions on behavioral decisions. For example, the technology acceptance model (TAM) proposes that individuals’ desire to accept a certain technology is determined by the degree to which it improves work performance and its ease of use (Davis, 1989; Tian et al., 2024). Furthermore, according to the equity implementation model (EIM), people’s concerns about the ratio of technical inputs to benefits and comparisons with the advantages obtained by others in society have a substantial influence on their adoption behavior (Joshi, 1991). Some scholars, however, have indicated that “utility maximization” does not always serve as a criterion for people’s actions, and the rational paradigm may not competently explain people’s decision-making behavior (Baron, 1994; Yang and Lester, 2008). For example, Parasuraman (2000) found that individuals’ perceived discomfort and insecurity regarding innovative technologies are important limiting factors in their adoption process, and the normative sociocultural pressures of adopting innovative technologies may also lead to resistance behavior (Migliore et al., 2022). However, few studies have examined the influence of irrational motivations and psychological mechanisms on health chatbot resistance behaviors. As such, the second research question of this study was to explore the psychological mechanisms behind people’s resistance to health chatbots.

**Figure 1:**
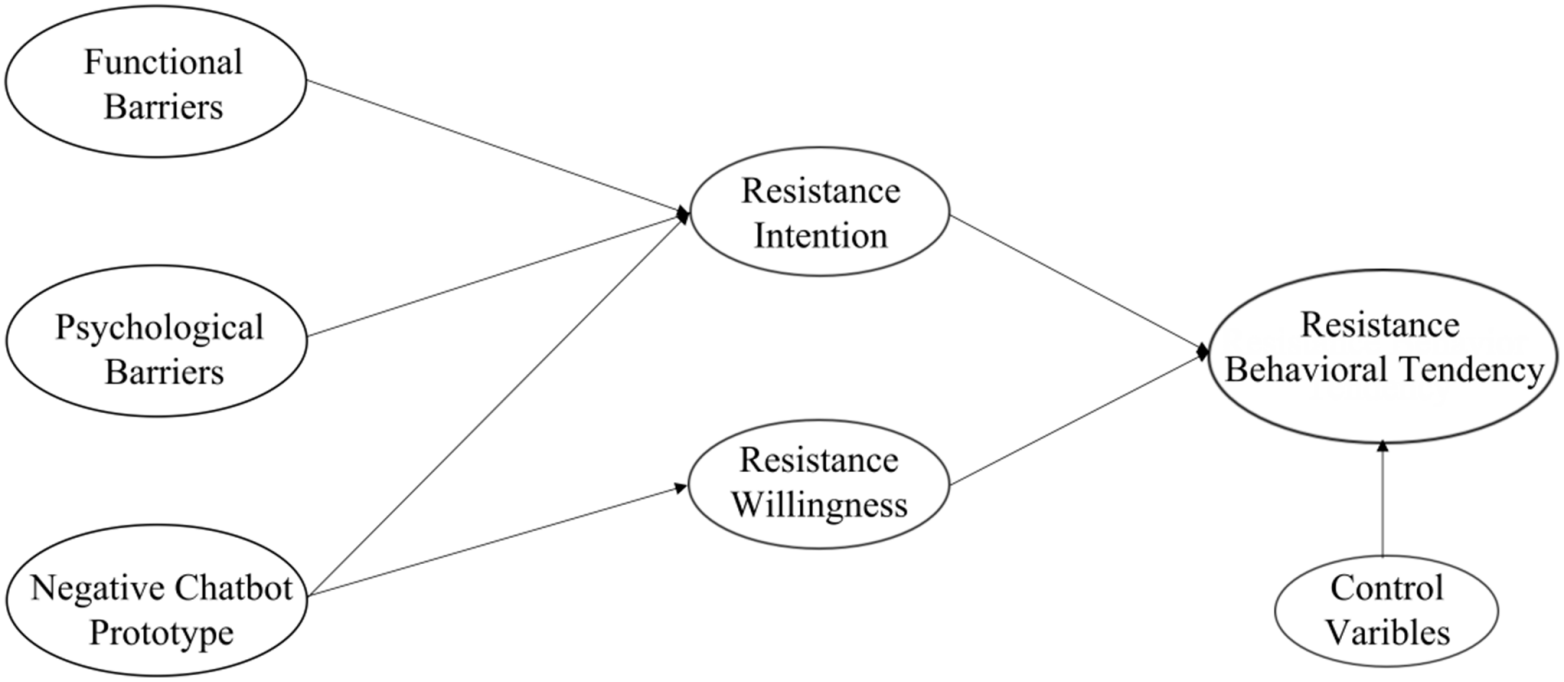
Conceptual framework.

To address the abovementioned research gaps, in this study, we first reviewed prior literature on the innovation resistance theory (IRT) and prototype willingness model (PWM); based on these theories, we then developed a parallel mediation model to investigate the antecedents of people’s resistance behavioral tendency of health chatbots, as well as the underlying psychological mechanisms. The conceptual framework of the study is illustrated in Figure 1. The current study contributes to the existing literature in the following ways. First, while numerous prior studies have examined attitudes toward AI healthcare technologies and motivations for their adoption (Esmaeilzadeh, 2020; Gao et al., 2020; Khanijahani et al., 2022), the current study contributes to the existing literature by investigating people’s behavior in resisting health chatbots and the underlying psychological mechanisms. Second, by identifying the rational and irrational factors influencing individuals’ resistance to health chatbots, this study advances the established literature’s comprehension of resistance behavior toward health chatbots. Finally, by combining the IRT and PWM, this study identifies the dual rational/irrational mediating mechanisms that influence people’s health chatbot resistance behavioral tendency and provides a valuable and insightful perspective for conducting future research on medical AI adoption behavior.

## 2 Literature review and hypothesis development

### 2.1 Innovation resistance theory

The IRT, initially proposed by Ram (1987), draws on the diffusion of innovation theory (DIT; Rogers and Adhikarya, 1979) and attempts to explain why people oppose innovation from a negative behavioral perspective. Individual resistance to innovation, according to the IRT, originates from changes in established behavioral patterns and the uncertainty aspect of innovation (Ram and Sheth, 1989). If innovation is likely to disrupt daily routines and conflict with established behavioral patterns and customs, individuals may refuse to utilize it and thus develop resistance behavior (Ram, 1987). Subsequently, Ram and Sheth (1989) revised the IRT by proposing that two particular barriers perceived by individuals when confronted with innovation, namely, functional and psychological barriers, result in their resistance behavioral tendency. Functional barriers refer to potential conflicts between individuals and innovations in terms of usage, value, and risk, whereas psychological barriers relate to the potential impact of individuals’ perceived innovation on established social beliefs, constituting tradition and image barriers (Kleijnen et al., 2009; Kaur et al., 2020).

The IRT provides a comprehensive operationalization framework for examining individual resistance to innovative technologies (Kleijnen et al., 2009). Previous research has demonstrated that functional and psychological barriers can significantly predict people’s resistance intentions and behaviors toward innovative technologies. For example, the IRT explains approximately 60% of the variance in people’s resistance to mobile payment technology (Kaur et al., 2020) and nearly 55% of the variance in their resistance to the online purchase of experience goods (Lian and Yen, 2013). Specifically, in terms of functional barriers, Prakash and Das (2022) discovered that the perceived value barriers, usage complexity, and privacy disclosure risks of digital contact-tracking apps can increase the intentions to resist such devices. Singh and Pandey (2024) also indicated that inefficient collaboration with AI devices is also a critical barrier to their usage. Yun and Park (2022), conversely, found that the reliability of chatbot service quality positively impacts users’ satisfaction and repurchase intention. Regarding psychological barriers, Chen et al. (2022) found that individuals’ perceived tradition barriers to changes in established ticketing habits brought about by mobile ticketing services were key predictors of resistance, and that those with a negative perception of online banking were more likely to display subsequent resistance (Kuisma et al., 2007).

Given that the IRT has previously demonstrated effective predictions of resistance intention and subsequent resistance behavior in innovative technologies such as mobile payments (Kaur et al., 2020), internet banking (Laukkanen et al., 2009), and smart home services (Hong et al., 2020), this study speculates that functional and psychological barriers of health chatbots are positively associated with people’s resistance intention. In a word, Individuals may be unwilling to engage with health chatbots if they believe that there are more barriers and risks involved than benefits. Similarly, when people have an adverse impression of the actual utility of health chatbots and perceive them as contradictory to their own healthcare-seeking norms, they may develop resistance intention to health chatbots. Accordingly, this study proposes the following research hypotheses:

*H1*: Functional barriers have a positive effect on health chatbot resistance intention.

*H2*: Psychological barriers have a positive effect on health chatbot resistance intention.

### 2.2 Prototype perception and resistance behavior

Prototypes are social images that represent individuals’ intuitive perceptions of the typical characteristics conveyed by engaging in certain social behaviors, such as the degree to which they evaluate behaviors including smoking (Piko et al., 2007), alcohol abuse (Norman et al., 2007), substance use (Wills et al., 2003), and risky selfies (Chen et al., 2019). In daily life, prototypes are commonly perceived as representations of a particular group that are easily identifiable and visible (Gibbons and Gerrard, 1995). Prototype perceptions of specific groups or social behaviors facilitate or inhibit individual behavioral tendencies (Thornton et al., 2002; Gerrard et al., 2008; Litt and Lewis, 2016; Lazuras et al., 2019). For example, adolescents who have negative prototype perceptions of smoking (e.g., it is “stupid”) significantly predict resistance to smoking (Piko et al., 2007). Conversely, if they perceived smoking as a positive prototype (e.g., it is “cool”), they were more likely to smoke (Gibbons and Gerrard, 1995). Thus, by adapting to, assimilating, or distancing themselves from specific prototypes, individuals can adopt behaviors that build a desired self-image or resist certain behaviors to avoid a socially unfavorable image (Gibbons et al., 1991; Gibbons and Gerrard, 1995).

Individual attitudes and subsequent behavioral tendencies are commonly thought to be influenced by prototypical similarity and favorability (Lane and Gibbons, 2007; Branley and Covey, 2018). Prototypical similarity is the degree of similarity between the individual’s perceived self and the prototype, and is usually assessed by the individual’s response to the question “How similar are you to the prototype?” (Gerrard et al., 2008). Prototypical favorability is considered to be an individual’s intuitive attitudinal evaluation toward a certain group or behavior, the assessment of which usually involves adjectival descriptors (Gibbons and Gerrard, 1995). For example, prototype favorability is usually measured by evaluating how certain behaviors are consistent with a series of adjectives such as “popular” or “unpleasant.” The more favorable individuals’ attitudes toward particular groups or objects, the greater their likelihood of joining the group or engaging in that behavior, and vice versa (Piko et al., 2007). Yokoi et al. (2021) discovered that the perceived low-value similarity of AI healthcare technologies led to a distrust of AI healthcare systems, and the degree of perceived anxiety and fear regarding health services may also lead to individual resistance behavior (Tsai et al., 2019).

Although initial research on the PWM relied solely on prototypical perceptions to explain behavioral willingness (Blanton et al., 1997; Gibbons et al., 1998), recent studies have shown that individuals’ prototypical perceptions can also explain behavioral intentions (Norman et al., 2007; Zimmermann and Sieverding, 2010). Further research on the effects of prototypical properties by Blanton et al. (2001) suggests that negative prototypical perceptions are more likely to lead to personal behavioral changes. In an investigation of teenage smoking resistance, it was observed that negative prototype perceptions were more likely to profoundly influence behavioral decisions than positive perceptions (Piko et al., 2007).

Based on the studies above, it can be inferred that if people have negative prototypical beliefs about health chatbots, such as “unsafe” and “unreliable,” their subsequent resistance intention and willingness to health chatbots are more likely to be substantial. Thus, this study speculates that a negative prototype perception regarding health chatbots may increase people’s resistance intention and willingness, and the following research hypotheses are proposed:

*H3a*: A negative prototype perception regarding health chatbots has a positive effect on resistance intention.

*H3b*: A negative prototype perception regarding health chatbots has a positive effect on resistance willingness.

### 2.3 Mediating role of resistance intention and resistance willingness

Previous studies have investigated the acceptance and resistance behaviors of individuals in the context of innovative medical technologies from a rational decision-making perspective (Tavares and Oliveira, 2018; Ye et al., 2019), generally concluding that individuals’ adoption behaviors toward medical technologies are the result of thoughtful deliberation (Deng et al., 2018; Wang et al., 2022). However, individuals’ decisions to accept healthcare innovations are not necessarily reasonable or logical. Irrational elements such as self-related emotions (Sun et al., 2023), social pressure (Jianxun et al., 2021), and specific sociocultural contexts (Hoque and Bao, 2015; Low et al., 2021) have also been found to have a significant impact on decisions to utilize digital health technology.

According to the PWM, “reasoned action” and “social reaction” constitute the two pathways through which individuals process information (Gibbons et al., 1998). “Reasoned action” is considered to be akin to the deductive pathway of the theory of reasoned action (TRA), which refers to people’s behavioral intention based on rational considerations and after thoroughly considering the consequences of a given behavior (Todd et al., 2016). For example, the perceived usefulness and usability of telemedicine technology are critical for promoting usage intentions and behaviors (Rho et al., 2014), whereas the greater the perceived performance and privacy risks of mobile physician procedures, the lower the adoption intentions (Klaver et al., 2021). Meanwhile, according to Todd et al. (2016), the “social reaction” pathway is dominated by irrational causes and is a behavioral reaction based on intuitive or heuristic elements. Thus, in contrast to behavioral intentions, which are built on rational decision-making, behavioral willingness represents reactive actions in response to a specific situational stimulus or social stress (Chen et al., 2019) and is more likely to be profoundly influenced by perceived prototypes (Hyde and White, 2010). For example, in a prior study investigating expert perspectives on the acceptance of chatbots for sexual and reproductive health (SRH) services, half the participants from the total sample expressed low acceptance because of the perceived unreliability of such devices (Nadarzynski et al., 2023).

Based on the aforementioned studies, this study suggests that there is a dual psychological process of resistance intention and resistance willingness behind people’s resistance behavioral tendency of health chatbots. Resistance intention refers to individuals’ taking action based on rational considerations before engaging in resistance behavior. Resistance willingness is recognized as a relatively emotional and impulsive behavioral tendency behind individuals’ resistance behavior (Gerrard et al., 2008; Todd et al., 2016), where individuals are more likely motivated by social pressure and ambiguous perceptions regarding the typical negative characteristics of a specific technology. In summary, individuals may follow both rational and irrational behavioral paths in the process of innovative medical technology utilization. Consequently, this study delineates the rational and irrational psychological processes behind individuals’ resistance behavior toward health chatbots and investigates the potential influence of two psychological mechanisms, resistance intention and resistance willingness, on their resistance behavioral tendency. Due to the functional and psychological barriers of the IRT stemming from the “rational person hypothesis,” it is emphasized that economic gains and cost savings will largely influence the likelihood of innovation resistance (Szmigin and Foxall, 1997). Therefore, this study speculates resistance intention mediates the relationships between functional barriers, psychological barriers, and resistance behavioral tendency, respectively. Furthermore, resistance intention and resistance willingness were hypothesized to mediate the relationship between negative prototype perceptions of health chatbots and resistance behavioral tendency. Accordingly, this study proposes the following hypotheses:

*H4*: Resistance intention and resistance willingness toward health chatbots have positive effects on resistance behavioral tendency.

*H5*: Functional barriers and resistance behavioral tendency are mediated by resistance intention toward health chatbots.

*H6*: Psychological barriers and resistance behavioral tendency are mediated by resistance intention toward health chatbots.

*H7*: A negative prototype perception regarding health chatbots and resistance behavioral tendency are mediated by resistance intention and resistance willingness toward health chatbots.

## 3 Methodology

### 3.1 Sample and procedure

To examine the research hypotheses, data were collected using *credamo* (www.credamo. com), a popular online questionnaire research platform in China; the respondents were provided extrinsic incentives to register for the survey (Zheng, 2023). By checking each participant’s IP address and limiting each device to a single response, the questionnaire system automatically ensured the validity of the answers. Following completion of the informed consent form, participants completed a self-report questionnaire about health chatbots through *credamo*. To ensure that each participant was fully informed about health chatbots and their usage scenarios, the respondents were initially provided with a description of what a health chatbot is and how it functions. Following this step, a total of 28 items were presented in the main component of the questionnaire to evaluate respondents’ views concerning functional barriers, psychological barriers, prototype perceptions, resistance intentions, resistance willingness, and resistance behavioral tendency of health chatbots. Finally, respondents were asked to provide demographic information including gender, age, education, income, residence, and experience with health chatbots. A total of 406 questionnaires were collected; however, eight individuals were excluded because they failed the attention check, and 398 participants qualified to form our research sample. The Ethics Committee of the School of Journalism and Communication, Jinan University (China; JNUSJC-2023-018) provided ethics approval for this study.

The sample size was determined following the criteria recommended by Kline (2023), which suggests that the ratio of the number of measured items to the number of participants should be at least 1:10. The 398 participants whose data were used for our analysis exceeded this sample size estimation and satisfied the academic recommendations. Table 1 presents the demographic information of the participants.

**Table 1:** Demographics information of respondents.

Demographic variables	Groups	Frequency	Percentage (%)
Gender			
	Male	144	36.18%
	Female	254	63.82%
Age			
	0–20	4	1.01%
	21–30	172	43.22%
	31–40	190	47.74%
	41–50	20	5.03%
	51–60	12	3.02%
Education			
	High School and below	6	1.51%
	Junior college	28	7.04%
	Bachelor’s degree	298	74.87%
	Master’s degree and above	66	16.58%
Income (¥)			
	0–2000	14	3.52%
	2001–6,000	60	15.08%
	6,001–10,000	154	38.69%
	10,001–150,000	78	19.60%
	More than 15,000	92	23.12%
Residence			
	Rural	17	4.27%
	Urban	381	95.73%
Health Chatbot Usage Experience			
	Yes	97	24.37%
	No	301	75.63%

### 3.2 Measures

All measuring instruments utilized in this study were checked by two professionals in this research field, who jointly translated the instruments into Chinese after discussing and resolving differences to improve the questionnaires’ clarity, reliability, and content validity. Twenty-three respondents, including two experts, were included in a pretest to ensure the semantic content of the items and logical structure of the questionnaires. The questionnaires’ content and structure were modified based on their feedback, as necessary. All items (Table 2) were evaluated using a 5-point Likert scale ranging from “1 = completely disagree” to “5 = completely agree.”

**Table 2:** Measurement model assessment.

Construct	Items	VIF	Loading	Cronbach’s α	rho_A	CR	AVE
Functional Barrier (FB)							
Usage barrier (UB) (Laukkanen, 2016)	Health chatbots are easy to use. (R) It’s convenient to use health chatbots. (R) Progress in health chatbots services is clear. (R) Using health chatbots for disease diagnosis easy. (R)	1.907 1.511 1.772 1.915	0.818 0.758 0.821 0.832	0.822	0.823	0.882	0.652
Value barrier (VB) (Laukkanen, 2016)	Compared to human doctors, health chatbots have no advantages for treating. Health chatbots will improve my ability to manage diseases. (R)	1.532 1.532	0.897 0.886	0.742	0.743	0.886	0.795
Risk barrier (RB) (Chakraborty et al., 2022)	My health information will be misused by the service provider of the health chatbots. Health chatbots will disclose my personal privacy data. Using health chatbots have some serious consequences that I cannot predict currently.	1.859 2.142 2.102	0.857 0.881 0.880	0.844	0.844	0.906	0.762
Psychological Barrier (PB)							
Tradition barrier (TB) (Laukkanen, 2016)	When I need medical treatment, I prefer the human doctor. When I need medical treatment, I prefer the health chatbots. (R)	1.791 1.791	0.913 0.912	0.798	0.798	0.908	0.832
Image barrier (IB) (Laukkanen, 2016)	I have the impression that health chatbots are too complex and useless. I have the impression that health chatbots are often difficult to use.	1.973 1.973	0.920 0.925	0.825	0.826	0.92	0.851
Negative Health Chatbot Prototype (NHCP) (Esmaeilzadeh, 2020; Wu et al., 2023)	Health chatbots are dangerous. Health chatbots are not to be trusted. Health chatbots are unreliable. Health chatbots will replace human doctors.	2.026 1.781 1.577 2.387	0.846 0.796 0.769 0.880	0.841	0.851	0.894	0.679
Resistance Intention (RI) (Van Gool et al., 2015)	I tend to resist health chatbots. I was likely to resist health chatbots. I do not think it’s impossible for me to resist health chatbots. I think I’ll resist health chatbots.	2.610 2.215 2.823 2.414	0.877 0.838 0.896 0.864	0.892	0.895	0.925	0.756
Resistance Willingness (RW) (Van Gool et al., 2015)	I will also resist health chatbots. Responding the same to resist health chatbots. Doing the same to resist health chatbots.	1.887 2.012 2.266	0.859 0.868 0.892	0.844	0.844	0.906	0.762
Resistance Behavioral Tendency (RBT) (Laumer et al., 2016)	I will not accept the recommendations of health chatbots. I will not cooperate with the health chatbots. I am opposed to the changes in medical practice brought by health chatbots. I disagree that health chatbots have changed the traditional way of treating diseases.	1.819 1.908 1.869 1.765	0.819 0.834 0.818 0.799	0.835	0.836	0.890	0.668

VIF, variance inflation factor; rho_A, Dijkstra-Henseler’s ρA; CR, composite reliability; AVE, average variance extracted. R, reverse scoring.

#### 3.2.1 Functional barriers

According to Ram and Sheth (1989), functional barriers are the constraints of innovative technologies that require changes in users’ established behavioral habits, norms, and traditions. They include three dimensions of individual perceptions relating to usage barriers, value barriers, and risk barriers regarding innovation. This study measured the perceived functional barriers of people’s resistance to health chatbots in terms of the three dimensions mentioned above.

*Usage Barriers (UB):* Usage barriers indicate the amount of effort required to comprehend and utilize innovative technologies, as well as the degree of change to existing usage routines and habits (Ram and Sheth, 1989). Four items derived from Laukkanen (2016) were used to evaluate individual assessments of usage barriers related to health chatbots; for example, “Health chatbots are easy to use” (Cronbach’s α = 0.823).

*Value Barriers (VB):* Value barriers are generated by inconsistencies between innovations and current value systems, particularly when there are inequitable advantages when adopting innovative technologies (Parasuraman and Grewal, 2000). Two items adapted from Laukkanen (2016) were used to evaluate individuals’ perceptions of value barriers related to health chatbots; for example, “Compared to human doctors, health chatbots have no advantages for treating” (Cronbach’s α = 0.742).

*Risk Barriers (RB):* Risk barriers are described as adoption barriers caused by users’ perceived uncertainty regarding innovative technologies (Marett et al., 2015). Three items derived from Chakraborty et al. (2022) were used to evaluate individuals’ risk perceptions of health chatbots; for example, “My health information will be misused by the service provider of the health chatbots” (Cronbach’s α = 0.844).

#### 3.2.2 Psychological barriers

Innovation may lead to psychological contradictions for individuals in some aspects, such as the impact of individuals’ technology utilization on their traditions and norms, and the perceived image barriers of innovation, which include the two dimensions of tradition barriers and image barriers (Ram and Sheth, 1989; Yu and Chantatub, 2015).

*Tradition Barriers (TB):* Tradition barriers are characterized by changes in existing user routines, culture, and behaviors, as well as social pressures linked to the application of innovation (Sadiq et al., 2021). Two items adapted from Laukkanen (2016) were used to evaluate individuals’ perceptions of tradition barriers related to health chatbots; for example, “When I need medical treatment, I prefer the human doctor” (Cronbach’s α = 0.798).

*Image Barriers (IB):* Image barriers refer to individuals’ negative impressions of innovation, focusing primarily on perceptions of the level of complexity of innovation utilization (Lian and Yen, 2014). Two items adapted from Laukkanen (2016) were used to evaluate individuals’ perceptions of image barriers related to health chatbots; for example, “I have the impression that health chatbots are too complex and useless” (Cronbach’s α = 0.825).

#### 3.2.3 Negative health Chatbot prototype

In line with the prototype perception literature (Gibbons and Gerrard, 1995; Lazuras et al., 2019) and existing public perception research on medical AI (Esmaeilzadeh, 2020; Wu et al., 2023), the participants were asked to self-report their perceptions of the typical risk characteristics of health chatbots. An item example is as follows: “Health chatbots are dangerous” (Cronbach’s α = 0.841).

#### 3.2.4 Resistance intention

Four items adapted from Van Gool et al. (2015) were used to evaluate individuals’ resistance intentions toward health chatbots. For example, an item is “I tend to resist health chatbots” (Cronbach’s α = 0.892).

#### 3.2.5 Resistance willingness

Based on the prototype perception literature (Gibbons and Gerrard, 1995; Van Gool et al., 2015), resistance willingness toward health chatbots was measured by asking individuals about their experience in a given scenario: “If in real life and the online world, you found yourself surrounded by people who were resisting health chatbots, what would you do?” The participants were asked to self-report their resistance willingness in response to three items based on this scenario; for example, “I will also resist health chatbots” (Cronbach’s α = 0.844).

#### 3.2.6 Resistance behavioral tendency

Three items adapted from Laumer et al. (2016) were utilized to evaluate individual resistance behavioral tendency of health chatbots. An item example is as follows: “I will not accept the recommendations of health chatbots” (Cronbach’s α = 0.835).

## 4 Results

Partial least squares structural equation modeling (PLS-SEM) was employed to examine the proposed research model. Compared to covariance-based structural equation modeling (CB-SEM), another important structural equation modeling method, PLS-SEM has flexibility in model construction, supports path estimation, and computes model parameters under non-normal distribution conditions (Hulland, 1999), This maximizes the explanatory power of endogenous variables, making it more appropriate for small and medium samples, as well as for studies targeting causal inference and predictiveness (Hair et al., 2019). Generally, PLS-SEM consists of two components: a measurement model used to examine the correlation between observable and latent variables and a structural model used to examine the correlation between exogenous and endogenous latent variables.

### 4.1 Common method bias

Cross-sectional surveys based on respondents’ self-reports may have a common method bias (CMB) issue (Podsakoff et al., 2003). This study first employed Harman’s single-factor technique to examine possible CMB, and the results revealed that the single factor contributed 33.29% of the total variance and did not exceed the 50% threshold (Chang et al., 2020). Second, the potential marker method was used to evaluate CMB, utilizing age as the marker variable (Li et al., 2023); the results showed that the correlation coefficient between the marker variable and other variables in our model did not exceed 0.3 (Lindell and Whitney, 2001). Finally, the collinearity diagnostics results among the explanatory variables revealed that the variance inflation factor (VIF) was less than 3.3 (Kock, 2015). The statistical indicators shown above imply that there was no CMB in this study.

### 4.2 Measurement model assessment

First, the measurement model was tested to examine the validity and reliability of the survey instruments. Table 2 shows that for all the instruments, the Cronbach’s α, Dijkstra-Henseler’s ρA, and composite reliability (CR) exceed 0.7, indicating acceptable internal reliability of the tools (Fornell and Larcker, 1981; Dijkstra and Henseler, 2015). Furthermore, the factor loadings of the instruments were all higher than the expected value of 0.7, and the average variance extracted (AVE) varied from 0.652 to 0.851, which is higher than the threshold value of 0.5 (Fornell and Larcker, 1981). Consequently, the convergent validity of the instruments was verified.

Second, the discriminant validity of the instruments was examined. As shown in Table 3, the HTMT ratio varied from 0.128 to 0.741 and did not exceed the 0.85 threshold, whereas the confidence interval of the HTMT ratio did not exceed 1.00 (Henseler et al., 2015). Table 4 indicates that the correlation coefficient between any two variables is less than 0.8, and the square root of the AVE exceeds the value of the correlation coefficient between the variables (Campbell and Fiske, 1959; Fornell and Larcker, 1981). These results suggest that the measurements passed the discriminant validity test.

**Table 3:** Discriminant validity of HTMT ratio and 95% confidence interval.

Construct	1	2	3	4	5	6	7	8	9
UB									
VB	0.659 [0.574; 0.749]								
RB	0.654 [0.566; 0.738]	0.714 [0.631; 0.797]							
TB	0.361 [0.253; 0.469]	0.277 [0.153; 0.401]	0.31 [0.193; 0.424]						
IB	0.368 [0.257; 0.481]	0.316 [0.196; 0.443]	0.306 [0.203; 0.412]	0.624 [0.543; 0.710]					
NHCP	0.253 [0.144; 0.374]	0.216 [0.111; 0.334]	0.251 [0.129; 0.368]	0.468 [0.362; 0.568]	0.462 [0.355; 0.557]				
RI	0.542 [0.455; 0.628]	0.678 [0.583; 0.77]	0.565 [0.481; 0.647]	0.41 [0.296; 0.516]	0.287 [0.188; 0.388]	0.342 [0.227; 0.449]			
RW	0.128 [0.078; 0.243]	0.142 [0.050; 0.26]	0.200 [0.093; 0.319]	0.408 [0.294; 0.523]	0.332 [0.23; 0.43]	0.741 [0.665; 0.814]	0.206 [0.101; 0.312]		
RBT	0.278 [0.167; 0.396]	0.269 [0.147; 0.398]	0.263 [0.149; 0.378]	0.641 [0.55; 0.725]	0.52 [0.425; 0.611]	0.537 [0.437; 0.628]	0.516 [0.421; 0.611]	0.638 [0.555; 0.717]	

HTMT, Heterotrait-monotrait; UB, Usage barriers; VB, Value barriers; RB, Risk barriers; TB, Tradition barriers; IB, Image barriers; NHCP, Negative health chatbot prototype; RI, Resistance intention; RW, Resistance willingness; RBT, Resistance behavioral tendency.

**Table 4:** The square root of AVE and correlation coefficient between variables.

Construct	1	2	3	4	5	6	7	8	9
UB	0.808								
VB	0.515	0.891							
RB	0.546	0.565	0.818						
TB	0.292	0.213	0.525	0.912					
IB	0.303	0.247	0.256	0.507	0.923				
NHCP	0.212	0.173	0.212	0.386	0.384	0.824			
RI	0.467	0.553	0.492	0.346	0.247	0.297	0.869		
RW	0.108	0.111	0.170	0.338	0.277	0.627	0.179	0.873	
RBT	0.229	0.213	0.221	0.525	0.432	0.454	0.447	0.536	0.818

AVE, average variance extracted; UB, Usage barriers; VB, Value barriers; RB, Risk barriers; TB, Tradition barriers; IB, Image barriers; NHCP, Negative health chatbot prototype; RI, Resistance intention; RW, Resistance willingness; RBT, Resistance behavioral tendency.

Finally, the standardized root mean square residual (SRMR) and d_ULS were assessed to evaluate the global model fit. The SRMR index of the proposed research model was 0.054, which is lower than the recommended threshold of 0.08. The d_ULS is expected to be lower than 0.95 (Tenenhaus et al., 2005), and this value of the proposed research model was 0.926. Being higher than the recommended index, this indicated that the overall degree of model fit met the research requirements.

### 4.3 Structural model assessment

Using the PLS-SEM algorithm and bootstrapping resampling procedure, this study evaluated the path coefficients and significance of the proposed model. The explained variance (R^2^) and effect size (*f*^2^) were also estimated to test the model’s explanatory power and actual efficacy, respectively. The R^2^ values for resistance intention, resistance willingness, and resistance behavioral tendency were 0.377, 0.391, and 0.429, respectively, each of which was higher than 0.19 (Purwanto, 2021). This indicated that the research model had good explanatory power. The *f*^2^ value was used to estimate whether the latent variables had substantial effects on the endogenous variables. Table 5 indicates that, *f*^2^ values range from 0.015 to 0.646; this clarifies that in the model, two paths have weak effects and four paths exceed the medium effect (higher than 0.15; Cohen, 1988).

**Table 5:** Hypothesis testing results.

Hypothesis	B	β	SE	T	LLCI-ULCI	*f^2^*	*p*
FB→RI	0.670	0.519	0.051	13.045	0.569; 0.771	0.376	0.000
PB→RI	0.111	0.112	0.041	2.691	0.029; 0.192	0.015	0.007
NHCP→RI	0.122	0.122	0.046	2.651	0.035; 0.216	0.019	0.008
NHCP→RW	0.759	0.627	0.047	15.983	0.669; 0.855	0.646	0.000
RI→RBT	0.295	0.336	0.034	8.760	0.232; 0.362	0.188	0.000
RW→RBT	0.315	0.432	0.032	9.788	0.255; 0.381	0.286	0.000

FB, Functional Barriers; PB, Psychological Barriers; NHCP, Negative health chatbot prototype; RI, Resistance intention; RW, Resistance willingness; RBT, Resistance behavioral tendency. f^2^, effect size; LLCI, lower limit confidence interval; ULCI, Upper limit Confidence Interval.

As shown in Table 5, the results of the path analysis indicate that functional barriers positively influenced resistance intention (*β* = 0.519, *p* < 0.001), thus H1 was supported. Additionally, psychological barriers also had a significant positive effect on resistance intention (*β* = 0.112, *p* = 0.007), thus, H2 was supported. A negative prototype perception regarding health chatbots also had a significant positive effect on both resistance intention (*β* = 0.122, *p* = 0.008) and resistance willingness (*β* = 0.627, *p* < 0.001); thus, H3a and H3b were supported. Finally, both resistance intention (*β* = 0.336, *p* < 0.001) and resistance willingness (*β* = 0.432, *p* < 0.001) were positive predictors of resistance behavioral tendency; thus, H4 was supported.

**Figure 2:**
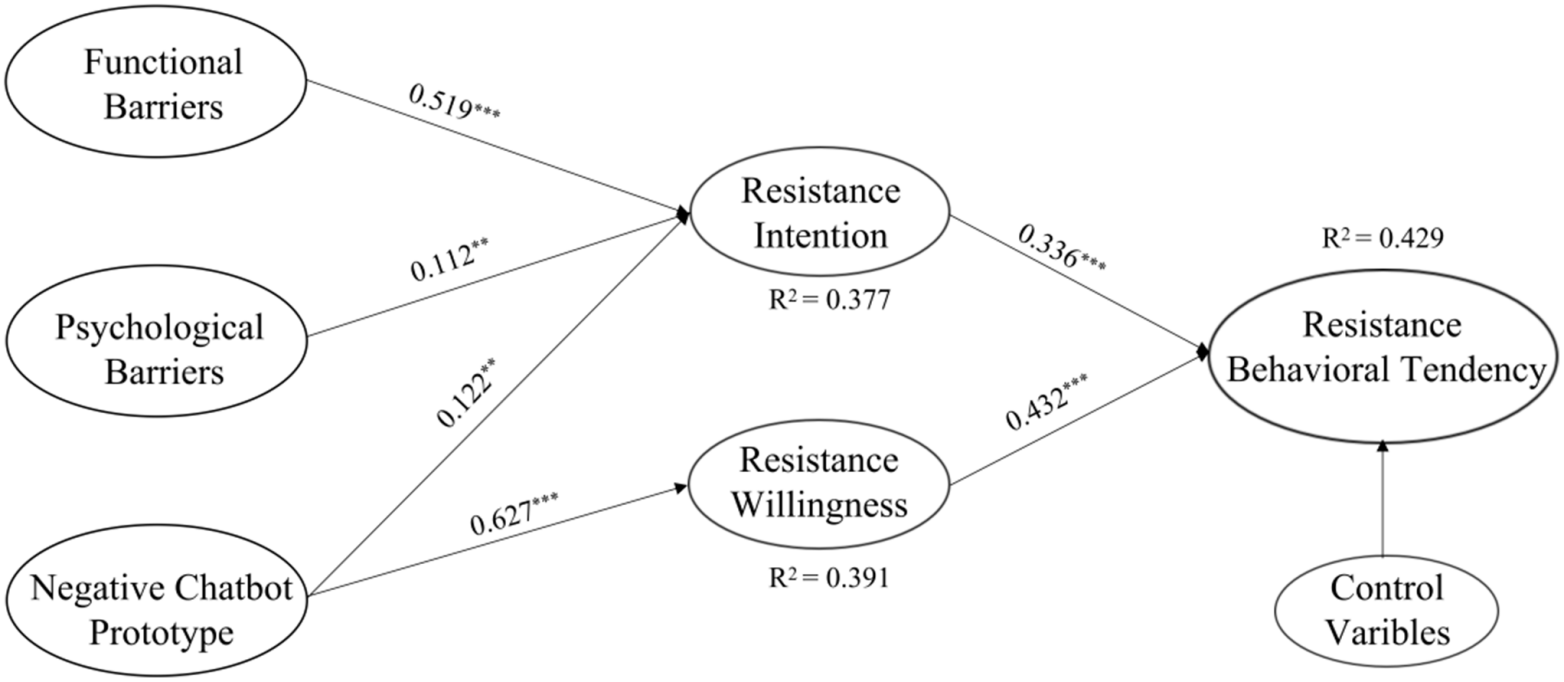
Structural model.

We also conducted a series of mediation analyses to examine the mediating role of resistance intention and resistance willingness between functional barriers, psychological barriers, a negative prototype perception regarding health chatbots, and resistance behavioral tendency. Table 6 indicates that resistance intention significantly mediated the link between functional barriers and resistance behavioral tendency (*β* = 0.174, CI [0.152; 0.247], *p <* 0.001). Thus, H5 was supported. Moreover, resistance intention (*β* = 0.038, CI [0.008; 0.062], *p* = 0.018) also had a significant mediating effect in the link between psychological barriers and resistance behavioral tendency. Thus, H6 was supported. Finally, resistance intention (*β* = 0.041, CI [0.010; 0.065], *p* = 0.010) and resistance willingness (*β* = 0.271, CI [0.191; 0.294], *p* < 0.001) also had significant mediating effects on the link between a negative prototype perception regarding health chatbots and resistance behavioral tendency. Thus, H7 was supported (see Figure 2).

**Table 6:** Mediation effects testing results.

Hypothesis	B	β	SE	T	LLCI-ULCI	*p*
FB→RI→RBT	0.197	0.174	0.025	8.018	0.152; 0.247	0.000
PB→RI→RBT	0.033	0.038	0.014	2.365	0.008; 0.062	0.018
NHCP→RI→RBT	0.036	0.041	0.014	2.586	0.010; 0.065	0.010
NHCP→RW→RBT	0.239	0.271	0.026	9.153	0.191; 0.294	0.000

FB, Functional Barriers; PB, Psychological Barriers; NHCP, Negative health chatbot prototype; RI, Resistance intention; RW, Resistance willingness; RBT, Resistance behavioral tendency. LLCI, lower limit confidence interval; ULCI, Upper limit Confidence Interval.

## 5 Discussion and implications

### 5.1 Discussion

This study aimed to examine the factors contributing to individuals’ resistance toward health chatbots, as well as the underlying psychological mechanisms, by constructing a parallel mediation model. Based on the theoretical frameworks of IRT and PWM, our results clarify the effects of functional barriers, psychological barriers, and negative prototype perceptions regarding health chatbots on resistance behavioral tendency, as well as the mediating roles played by resistance intention and resistance willingness between their linkages.

Consistent with prior studies conducted in the domain of innovation resistance (Sadiq et al., 2021; Cham et al., 2022; Friedman and Ormiston, 2022), this study revealed that perceived functional and psychological barriers also exerted a significant positive influence on individuals’ resistance intention to health chatbots. Moreover, according to the path coefficients of the findings, we found that functional barriers of health chatbots have a greater positive impact on people’s resistance intention and behavior than psychological barriers. This conclusion is similar to that of prior studies, such as Kautish et al. (2023), who found that functional barriers to telemedicine apps play a more predictable role in users’ purchase resistance intentions. Furthermore, Our results demonstrate that people’s negative prototype perception regarding health chatbots, such as their being “dangerous” and “untrustworthy,” significantly influence their resistance intention, resistance willingness, and resistance behavioral tendency. This suggests that people’s heuristic perceptions of the negative images and risk beliefs concerning health chatbots are important determinants of their resistance behavioral tendency. This finding responds to a previous research question on whether people’s negative psychological perceptions of healthcare AI affect their subsequent usage intentions and behaviors (Schepman and Rodway, 2020; Jussupow et al., 2022). Specifically, according to the path coefficients, a negative prototype perception regarding health chatbots had a greater impact on resistance behavioral tendency through resistance willingness than resistance intention. This is consistent with previous research, which found that prototypical perceptions influence individual behaviors through behavioral willingness rather than behavioral intentions in behaviors such as smoking (Gerrard et al., 2005) and alcohol abuse (Davies et al., 2016; Gibbons et al., 2016).

Drawing on the PWM, this study reveals the dual rational/irrational mediating mechanisms underlying people’s resistance to health chatbots. In particular, this study demonstrates that individuals’ perceived functional and psychological barriers may significantly influence their resistance intention, thereby increasing the likelihood of subsequent resistance behavioral tendency. Similarly, negative prototypes regarding health chatbots may increase resistance behavioral tendency through resistance intention and resistance willingness. Importantly, our results indicate that negative prototype perceptions regarding health chatbots have a greater impact on individuals’ resistance willingness and their subsequent resistance behavioral tendency than functional and psychological barriers. In summary, when confronted with irrational factors such as social pressure and intuitive negative cues, people are more likely to reject health chatbots. This is consistent with previous research by Sun et al. (2023), who discovered that the presence of emotional disgust toward smartphone apps reduced individuals’ adoption intentions. This result reaffirms the prior finding that prototype perceptions have a greater influence through behavioral willingness, and thus impact individual behavior (Myklestad and Rise, 2007; Abedini et al., 2014; Elliott et al., 2017). Fiske et al. (2019) explained that since people do not integrate AI devices into their real lives, their ambiguous perceptions arising from their lack of specific knowledge can significantly affect the perceived risks of AI technologies (Dwivedi et al., 2021), and may lead to their refusal to utilize health chatbots.

### 5.2 Implications

#### 5.2.1 Theoretical implications

By constructing a comprehensive model that includes rational and irrational psychological pathways to health chatbot resistance, this study contributes theoretically to the existing literature in the following ways. First, it enriches existing research on people’s acceptance behavior toward health chatbots. Previous studies have focused on investigating individuals’ attitudes toward health chatbots (Palanica et al., 2019), adoption motivations (Nadarzynski et al., 2019; Zhu et al., 2022), and psychological processes of adoption (Chang et al., 2022), with the aim of exploring ways to facilitate people’s adoption behavior in the context of medical AI technologies. However, identifying the factors that lead to people’s resistance to medical AI technology is a critical component in discovering ways to promote people’s adoption behaviors. This study systematically and empirically explored the factors and psychological mechanisms that influence people’s resistance to health chatbots by constructing a parallel mediation model. The study extends our understanding of individuals’ acceptance behaviors toward medical AI technologies from the perspective of their formative resistance behavioral tendency. Second, by combining the IRT and PWM, this study enriches existing literature on the antecedents and psychological pathways of individuals’ resistance to health chatbots. Prior research has primarily emphasized the impact of rational considerations such as acceptability (Boucher et al., 2021), perceived utility (Nadarzynski et al., 2019), and performance expectancy (Huang et al., 2021), on individuals’ health chatbot adoption behavior. This study focused on the effect of individuals’ direct heuristic negative prototype perceptions regarding health chatbots on resistance willingness and subsequent behavior, revealing that the irrational paths driven by negative prototype perceptions have a more profound influence on individuals’ resistance willingness and behavior toward health chatbots, providing valuable theoretical references for conducting future research on medical AI resistance behavior.

#### 5.2.2 Practical implications

This study also reveals some practical insights that can contribute to the development of interventions for addressing people’s resistance to health chatbots. First, our findings suggest that individuals’ perceived functional barriers to health chatbots can significantly influence their resistance intentions and behaviors. Therefore, designing more convenient and relatively user-friendly health chatbots may be the way forward. As noted by Lee et al. (2020), improving the interactivity and entertainment of AI devices in healthcare may help reduce communication barriers between users and AI devices, thus increasing the acceptance of health chatbots. In addition, service feedback mechanisms for health chatbots should be established and adequately evaluated to optimize the devices, which in turn would reduce the perceived complexity of health chatbots and actual usage difficulty. Second, this study found that individuals’ psychological barriers to health chatbots also significantly impact resistance intention as well as subsequent resistance behavioral tendency. Thus, future designers of health chatbots should consider the important influence of psychological barriers on resistance behavioral tendency. Accordingly, health chatbot providers should design products and services that are more applicable to people’s daily lives and decrease the degree of disruption to their established routines. Furthermore, offline health chatbot experience programs should be established to enhance people’s sense of security in utilizing health chatbots and encourage the acceptance of innovative medical AI technologies. The necessary knowledge about health chatbots and their advantages should be increased to reverse the possible negative perception of health chatbots and reduce individuals’ psychological discomfort in their adoption process (Röth and Spieth, 2019). Finally, our findings highlighted the significant impact of individuals’ negative prototype perceptions regarding health chatbots on their resistance behavioral tendency. Therefore, it is crucial to eliminate people’s instinctive negative views of health chatbots for their social popularization. Health chatbot providers, in particular, should utilize influential media channels to continuously disseminate information regarding health chatbots’ scientific utility to address asymmetric perceptions and promote an objective understanding of this technology. Moreover, scientific facts about health chatbots, such as functioning principles, utilization scenarios, and essential precautions, should be popularized by media outlets to reverse negative prototypical perceptions about health chatbots and support rational views and assessments regarding this technology.

## 6 Limitations and future research

The present study had some limitations. First, the data for this study were derived from a cross-sectional survey based on self-reports; future research could use experimental methods to acquire causal insights or conduct a longitudinal tracking survey to construct a more dynamic model that explores the evolution of resistance attitudes and behaviors. Second, although this study has demonstrated the influence of factors such as functional barriers, psychological barriers, and negative prototype perceptions regarding health chatbots on resistance behavioral tendency, the influences of innovation resistance are commonly grounded in specific scenarios (Claudy et al., 2015). Thus, it would be valuable for future studies to incorporate in-depth interviews as well as qualitative research methodologies such as grounded theory to obtain more comprehensive results on the impact of health chatbots on individuals. Third, the measurement of resistance behavioral tendency in this study may not strictly represent actual resistance behavior. It is recommended that future research adopt more direct methods to measure people’s actual resistance behavior toward health chatbots. Fourth, consistent with prior research, the current study investigated resistance psychology and behavior primarily from the perspective of individual perceptions, attitudes, and behaviors. However, the factors influencing individual resistance to innovative technologies are diverse (Talwar et al., 2020; Dhir et al., 2021). For example, a recent study confirmed that user emotions impact innovation evaluation and subsequent resistance behavior (Castro et al., 2020). Therefore, future research should consider the effects of factors such as individual emotions, cultural context, and social circumstances on individuals’ resistance behaviors.

## 7 Conclusion

The popularization of AI in healthcare depends on the population’s acceptance of related technologies, and overcoming individual resistance to AI healthcare technologies such as health chatbots is crucial for their diffusion (Tran et al., 2019; Gaczek et al., 2023). Based on the IRT and PWM, this study investigated the effects of functional barriers, psychological barriers, and negative prototypical perceptions regarding health chatbots on resistance behavioral tendency and further identified the mediating roles of resistance intention and resistance willingness between their associations. The results indicated that resistance intention mediated the relationship between functional barriers, psychological barriers, and resistance behavioral tendency, respectively. Furthermore, The relationship between negative prototype perceptions and resistance behavioral tendency was mediated by resistance intention and resistance willingness. Importantly, the present study found that negative prototypical perceptions were more predictive of resistance behavioral tendency than functional and psychological barriers. This study empirically demonstrates the influence of the dual psychological mechanisms of rationality and irrationality behind individuals’ resistance to health chatbots, expanding knowledge on resistance behaviors toward health chatbots and recommending ways to overcome this resistance through tailored interventions.

## Data availability statement

The datasets presented in this study can be found in online repositories. The names of the repository/repositories and accession number(s) can be found at: https://osf.io/d8mvu/.

## Ethics statement

The studies involving humans were approved by the Ethics Committee of the School of Journalism and Communication, Jinan University (China) (JNUSJC-2023-018). The studies were conducted in accordance with the local legislation and institutional requirements. The participants provided their written informed consent to participate in this study.

## Author contributions

XZ: Conceptualization, Data curation, Formal analysis, Methodology, Supervision, Writing – original draft, Writing – review & editing. YN: Formal analysis, Writing – original draft, Writing – review & editing. KL: Data curation, Funding acquisition, Project administration, Writing – review & editing. GL: Conceptualization, Formal analysis, Funding acquisition, Methodology, Supervision, Writing – original draft, Writing – review & editing.
